# A curious coincidence: mosquito biodiversity and the limits of the Japanese encephalitis virus in Australasia

**DOI:** 10.1186/1471-2148-7-100

**Published:** 2007-06-29

**Authors:** Stéphane Hemmerter, Jan Šlapeta, Andrew F van den Hurk, Robert D Cooper, Peter I Whelan, Richard C Russell, Cheryl A Johansen, Nigel W Beebe

**Affiliations:** 1 Institute for the Biotechnology of Infectious Diseases, University of Technology, Sydney, 1 Broadway, Ultimo, 2007, New South Wales, Australia; 2 Department of Microbiology and Parasitology, School of Molecular and Microbial Sciences, University of Queensland, St. Lucia, 4072, Queensland, Australia; 3 Australian Army Malaria Institute, Gallipoli Barracks, Enoggera, 4052, Queensland, Australia; 4 Medical Entomology, Centre for Disease Control, Department of Health and Community Services, Darwin, 0810, Northern Territory, Australia; 5 Department of Medical Entomology, University of Sydney and ICPMR, Westmead Hospital, Westmead, 2145, New South Wales, Australia; 6 Arbovirus Surveillance and Research Laboratory, School of Biomedical, Biomolecular and Chemical Sciences, University of Western Australia, Nedlands, 6009, Western Australia, Australia; 7 Faculty of Veterinary Science – B14, University of Sydney, New South Wales, 2006, Australia

## Abstract

**Background:**

The mosquito *Culex annulirostris *Skuse (Diptera: Culicidae) is the major vector of endemic arboviruses in Australia and is also responsible for the establishment of the Japanese encephalitis virus (JEV) in southern Papua New Guinea (PNG) as well as its incursions into northern Australia. Papua New Guinea and mainland Australia are separated by a small stretch of water, the Torres Strait, and its islands. While there has been regular JEV activity on these islands, JEV has not established on mainland Australia despite an abundance of *Cx. annulirostris *and porcine amplifying hosts. Despite the public health significance of this mosquito and the fact that its adults show overlapping morphology with close relative *Cx. palpalis *Taylor, its evolution and genetic structure remain undetermined. We address a hypothesis that there is significant genetic diversity in *Cx. annulirostris *and that the identification of this diversity will shed light on the paradox that JEV can cycle on an island 70 km from mainland Australia while not establishing in Australia itself.

**Results:**

We sequenced 538 bp of the mitochondrial *DNA cytochrome oxidase I *gene from 273 individuals collected from 43 localities in Australia and the southwest Pacific region to describe the phylogeography of *Cx. annulirostris *and its sister species *Cx. palpalis*. Maximum Likelihood and Bayesian analyses reveal supporting evidence for multiple divergent lineages that display geographic restriction. *Culex palpalis *contained three divergent lineages geographically restricted to southern Australia, northern Australia and Papua New Guinea (PNG). *Culex annulirostris *contained five geographically restricted divergent lineages, with one lineage restricted to the Solomon Islands and two identified mainly within Australia while two other lineages showed distributions in PNG and the Torres Strait Islands with a southern limit at the top of Australia's Cape York Peninsula.

**Conclusion:**

The existence of divergent mitochondrial lineages within *Cx. annulirostris *and *Cx. palpalis *helps explain the difficulty of using adult morphology to identify *Cx. annulirostris *and its ecological diversity. Notably, the southern limit of the PNG lineages of *Cx. annulirostris *coincides exactly with the current southern limit of JEV activity in Australasia suggesting that variation in these COI lineages may be the key to why JEV has not yet established yet on mainland Australia.

## Background

**Figure 1:**
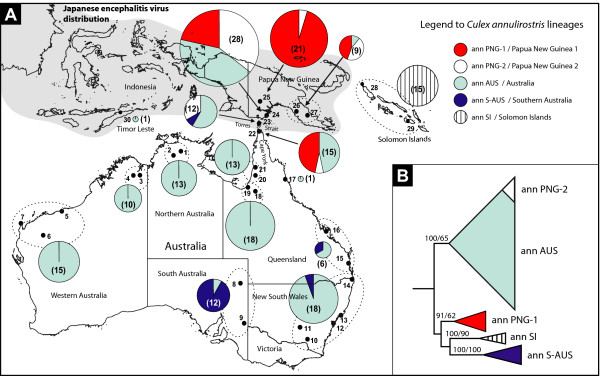
(A)** Map of collection sites in Australasia with proportional distribution of *Cx. annulirostris COI *lineages**. Collection sites are indicated 1–30 (for locality details see Table 1, Supplementary see Additional file 1 and 2). Pie chart graphs indicate the distributional frequency of *Cx. annulirostris *haplotypes representing the five identified mtDNA *COI *lineages. The size of the pie charts' segments is proportional to the number of mosquitoes identified as *Cx. annulirostris*, which is also indicated in brackets. Haplotype a51 from the laboratory colony at AMI in Queensland, Australia, is not included. (B) **Summarized Bayesian phylogenetic tree**. Phylogenetic tree of *Cx. annulirostris *showing 134 *COI *haplotypes compressed into the 5 different lineages of *Cx. annulirostris*. Branch support is Bayesian posterior probability/ML bootstraps (expanded in Figure 2). Haplotypes belonging to *Cx. sitiens *were used as an outgroup.

Northern Australia has the ideal conditions for the establishment of the Japanese encephalitis virus (JEV), given its dual abundance of both *Culex annulirostris *Skuse – a vector identified as transmitting JEV in the region [1,2] – and the feral pig populations that act as principal amplifying hosts for the virus's transmission to humans [3]. Over the last decade JEV has become endemic in Papua New Guinea (PNG), and now cycles yearly on islands in the Torres Strait 70 km from mainland Australia (see Figure 1; [4]). It has appeared twice on Australia's Cape York since 1998, but in each case the virus did not establish [4,5].

Given these conditions, the failure of JEV to establish itself on the Australian continent over the past decade has perplexed researchers. Suggested explanations for this have included the presence of alternative blood-meal hosts and competition with antigenically related arboviruses for susceptible vertebrate hosts [5]. But another possibility is that unrecognized species or population variation of this vector throughout southern PNG and northern Australia itself may limit the establishment of JEV. Are there differences between the *Cx. annulirostris *populations that exist where JEV occurs in PNG and the Torres Strait, and in mainland Australia that work to contain rather than spread JEV throughout this region? As this first unravelling of the genetic diversity of this species and its closely related sister species suggests, population variation around the southern limit of JEV may in fact restrict the movement of an arbovirus.

Although mosquitoes are the vectors of pathogens that cause significant human disease, their genetic diversity remains poorly understood. In our region of Australasia (Australia and the southwest Pacific) the closely related mosquito species *Cx. annulirostris*,* Cx. palpalis *Taylor and the coastally restricted *Cx. sitiens *Wiedemann are abundant with distributions in Australia, Papua New Guinea (PNG) and the Solomon Islands [6]. The morphospecies *Cx. annulirostris *transmits exotic JEV and is also Australia's principal vector of endemic arboviruses that cause human disease, including Ross River virus, Barmah Forest virus, Murray Valley encephalitis virus and Kunjin virus (a subtype of West Nile Virus) [1,3,7-9]]. The potential establishment of JEV in northern Australia is of serious concern to public health officials in Australia [9], as this virus, which was restricted to Southeast Asia, is responsible for an estimated 30,000–50,000 cases there annually [10]. Yet despite regular JEV activity in the Torres Strait and seroconversions of sentinel animals in 1998 and 2002 on Australia's Cape York, there is no evidence that JEV has become enzootic on the Australian mainland [5].

Overlapping morphology exists between these *Culex *taxa and allozyme and PCR-based procedures, previously developed to separate these mosquitoes, have suggested the presence of cryptic species within these morphospecies [11,12]. In this study we have sequenced the *cytochrome oxidase subunit I gene *(*COI*) to look at the genetic structure, evolution and distribution of *Cx. annulirostis, Cx. palpalis *and *Cx. sitiens *collected in Australasia. We evaluate this DNA barcoding marker in a phylogenetic framework to examine the genetic diversity of three *Culex *morphospecies, one of which is the most important arbovirus vector in Australasia.

We present sequence data generated from 273 mosquitoes that support the distinction of the three morphospecies, as well as the recognition of multiple divergent lineages. The phylogeographic pattern is most complex in *Cx. annulirostris *with two distinct paraphyletic lineages recognized within Australia – both independently related to the PNG lineages. This data provides phylogenetic support for a recent historical connection of mosquito populations in Australia and PNG that will facilitate more rational arbovirus competency experiments and better surveillance for these mosquitoes in the future. Importantly, the southern limit of the two PNG *Cx. annulirostris *lineages equates exactly to the current southern limit of JEV activity in the Australasian region.

## Results

### Mosquito identification: morphology versus rDNA ITS1 and COI

There was 10% incongruity between the ITS1 diagnostic results and the morphology of 218 field collected adults (see Additional file 1). For *Cx. annulirostris*, correct morphological identification occurred 95.6% of the time (8 individuals out of 180 were incorrectly identified as *Cx. palpalis*), 95.5% for *Cx. sitiens *(1 out of 22 individuals was incorrectly identified as *Cx. annulirostris*) and 62.5% for *Cx. palpalis *(14 of 40 were incorrectly identified as *Cx. annulirostris *and one as *Cx. sitiens*). These results confirm the difficulty distinguishing *Cx. palpalis *from *Cx. annulirostris *and highlight the fact that *Cx. palpalis *is probably underrepresented in studies that use morphology only for species discrimination.

Of 182 different *COI *haplotypes found in 273 field-collected mosquitoes – comprising 28 *Cx. sitiens*, 208 *Cx. annulirostris *and 37 *Cx. palpalis *– there was 100% agreement between the ITS1 diagnostic and the *COI *sequence grouping of *Culex *spp. The final sequence alignment (see Additional file 3) was 538 nucleotides of which 77% (414 nt) were constant, 19% (100 nt) were parsimony informative and the remaining 4% (24 nt) represented unique singletons. The majority (89%) of the parsimony informative sites were detected at the 3rd codon position, compared with 11% at the 1st codon position and none in the 2nd position. A pronounced AT-bias was observed at the 3rd codon position and when the three codons were examined separately, differences in nucleotide compositions were observed. For example, at the first codon position, guanine was represented 30.6% of the time, compared to 15.7% in the second codon position and 2.4% in the third codon position, suggesting that significant differences in the model of sequence evolution between three codon positions are required. To counterbalance the effect of discrepancy between codon position and nucleotide diversity, a GTR model (which allows for the variation of base frequencies and unique probability for each of the six possible substitution classes) was selected in agreement with ModelTest 3.6 [13] for the Maximum Likelihood analyses. The complex model of substitution including gamma distribution and invariant sites (GTR+Γ+I) was selected for the full alignment by the hierarchical likelihood ratio test (LRT) as well as the Akaike information criterion (AIC). Moreover, the dataset for the Bayesian analysis was separated into partitions corresponding to first, second and third codon positions, thus allowing application of independent parameters for these partitions in order to better fit the model to the dataset.

The translation of the nucleotide sequence into an amino acid sequence, using the invertebrate genetic code, showed identical amino acid sequences for all considered haplotypes in the three species, except for a single amino acid valine substituted to leucine in haplotype a126 (DQ673707) of *Cx. annulirostris *collected in PNG. Both valine and leucine are similar in their chemical properties, being non-charged and hydrophobic. This mutation has been verified using three independent PCR amplifications and direct bidirectional sequencing.

**Figure 2:**
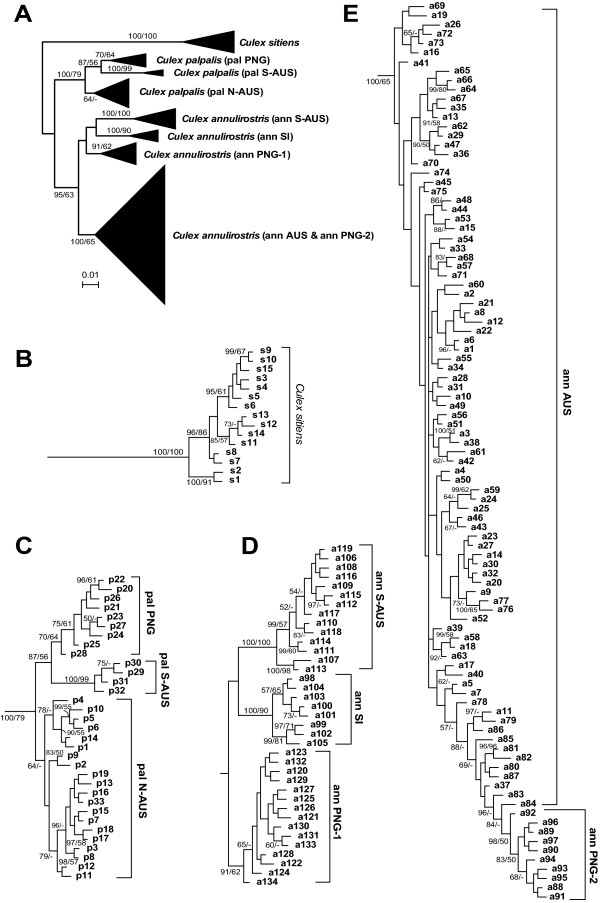
**Phylogenetic tree of *Culex *spp. based on *COI *gene sequence**. (A) Summarized Bayesian phylogenetic tree with 182 total *COI *haplotypes. *Culex sitiens *were used as an outgroup. (B) Expanded subtree of 15 haplotypes from 28 *Cx. sitiens*. (C) Expanded subtree of 33 haplotypes from 37 *Cx. palpalis *individuals. (D, E) Expanded subtree of 134 haplotypes from 208 *Cx. annulirostris *individuals. Bayesian tree was reconstructed based on nucleotide sequence alignment of 538 aligned coding positions using MrBayes 3.1.2 with a mixed nucleotide model. Bayesian posterior probabilities/ML bootstrap support values calculated with PhyML 2.4.4 (500 replicates) are shown (>50/50%). For details about phylogenetic reconstruction see Materials and Methods.

Both the Maximum Likelihood and Bayesian methods show that the *COI *was able to discriminate the three taxa and confirmed all ITS1 identifications as well as several divergent lineages within *Cx. annulirostris *and *Cx. palpalis *(Figure 2 and see Additional file 1). Both phylogenetic methods produced the same tree with Maximum Likelihood showing lower branch support.

*Culex sitiens *was the most divergent taxon, showing little geographic structure; the same haplotypes (s9, s8) occurred on both the east and west coasts of Australia. The haplotypes (s11–s14) from PNG do form a clade within the Australian grouping (s1–10, s15), but as only 26 individuals were assessed little can be interpreted from this.

Both phylogenetic methods support the division of *Cx. palpalis *into three divergent lineages (pal-N-AUS, pal-S-AUS and pal-PNG) that show no geographic overlap (see Additional file 2). The pal-PNG lineage was restricted to PNG and the Torres Strait; the pal-N-AUS lineage was restricted to northern Australia; and the pal-S-AUS was found only in southern Australia.

*Culex annulirostris *comprised four distinct lineages (ann-PNG1, ann-SI, ann-S-AUS and ann-AUS) with a fifth sublineage identified within the ann-AUS lineage (ann-PNG2) in Figures 1 and 2 due partly to the restricted distribution of this clade, which is the same as ann-PNG1. These lineages showed clear geographic structure (Figure 1). The ann-AUS lineage exists throughout Australia and southern PNG while the ann-S-AUS lineage appears to be more abundant in southern Australia making up 92% (11/12 mosquitoes) of collections in South Australia (sites 8 and 9) and also appearing sporadically along the east coast of Australia (single individuals identified at sites 15, 16 and 23). The PNG lineages ann-PNG1 and ann-PNG2 accounted for 76% of *Cx. annulirostris *in PNG (44/58 mosquitoes) with the Torres Strait and the top of Queensland's Cape York Peninsula representing their southern limit (sites 23 and 22). A fifth lineage ann-SI occurred only in the Solomon Islands (sites 28 and 29).

Haplotype diversity was very high in all lineages with most lineages showing values above 0.85 (Table 1). Apart from the major lineages described above and the sublineage ann-PNG2, no phylogeographic structure was found among the remaining haplotypes.

**Table 1:** Molecular diversity of haplotypes (nucleotides and haplotypes diversity)

**Mosquito species "mtDNA lineage"**	***N ***	**No. haplotypes**	**Nucleotide diversity (SD)**	**Haplotype diversity (SD)**
*Culex annulirostris *"ann AUS"	122	73	0.0080 (0.0005)	0.954 (0.014)
*Culex annulirostris *"ann PNG-1"	37	15	0.0022 (0.0003)	0.787 (0.061)
*Culex annulirostris *"ann PNG-2"	19	10	0.0025 (0.0004)	0.854 (0.061)
*Culex annulirostris *"ann S-AUS"	15	14	0.0068 (0.0012)	0.990 (0.028)
*Culex annulirostris *"ann SI"	15	8	0.0068 (0.0008)	0.848 (0.071)
*Culex palpalis *"pal N-AUS"	23	17	0.0072 (0.0008)	0.960 (0.027)
*Culex palpalis *"pal S-AUS"	4	4	0.0056 (0.0012)	1.000 (0.177)
*Culex palpalis *"pal PNG"	10	9	0.0065 (0.0008)	0.978 (0.054)
*Culex sitiens*	28	13	0.0077 (0.0015)	0.881 (0.041)

SD – standard deviations; N – sample size, number of mosquitoes.

### Genetic relationships within and between identified COI lineages

**Figure 3:**
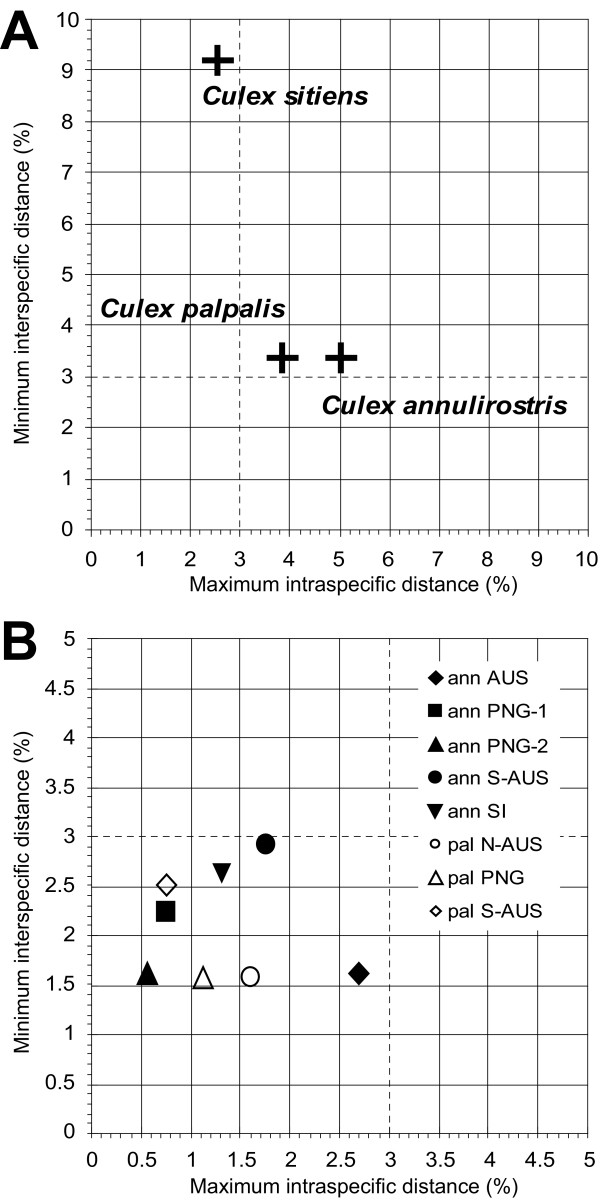
**Intraspecific vs. interspecific distance of *COI *sequence from *Cx. annulirostris*,*Cx. palpalis *and *Cx. sitiens***. (A) Morphological taxa maximum intraspecific distance was compared to the minimum interspecific congeneric difference (Kimura 2 distance). (B) The morphological taxa were divided into several proposed lineages and their maximum intra-lineage distance was compared to the minimum inter-lineage difference. For both graphs the 3% threshold is highlighted, dividing the graph into four quadrants that represent different categories of "species" [15]: top left – species concordant with current taxonomy; top right – probable composite species, *i.e*. candidates for taxonomic split; bottom left – species that have undergone recent divergence, hybridization, or synonymy; bottom right – probable specimen misidentification.

To evaluate the effectiveness of the barcoding methodology for identifying species biodiversity, we assessed the inter- and intraspecies divergence before and after the split of *Cx. annulirostis *and *Cx. palpalis *into their eight different lineages. For each population (species or lineages) we compared their minimum distance to a congener with the maximum divergence within each population (Figure 3). A threshold value of 3% was used to separate the graph into four quadrants representing the different categories of species [14,15]. The top right quadrant represents a high intra- and interspecific diversity suggesting that cryptic species are present, and the top left quadrant indicates high interspecific and low intraspecific diversity suggesting no cryptic species. The morphospecies *Cx. annulirostris *and *Cx. palpalis *appear in the top right quadrant (Figure 3A), indicating high levels of genetic diversity within and between species and suggesting these as candidates for a taxonomic split [15]. The presence of *Cx. sitiens *in the top left quadrant of Figure 3A indicates that it did not display sufficient within-species variation to generate doubt as to whether it was a single biological entity. When the five lineages of *Cx. annulirostris *and the three lineages of *Cx. palpalis*, identified through phylogenetic inference, were reanalyzed, all eight lineages appeared in the bottom left quadrant (Figure 3B), suggesting recently diverged populations and/or newly emerged species [15].

### Alternative tree topologies

**Figure 4:**
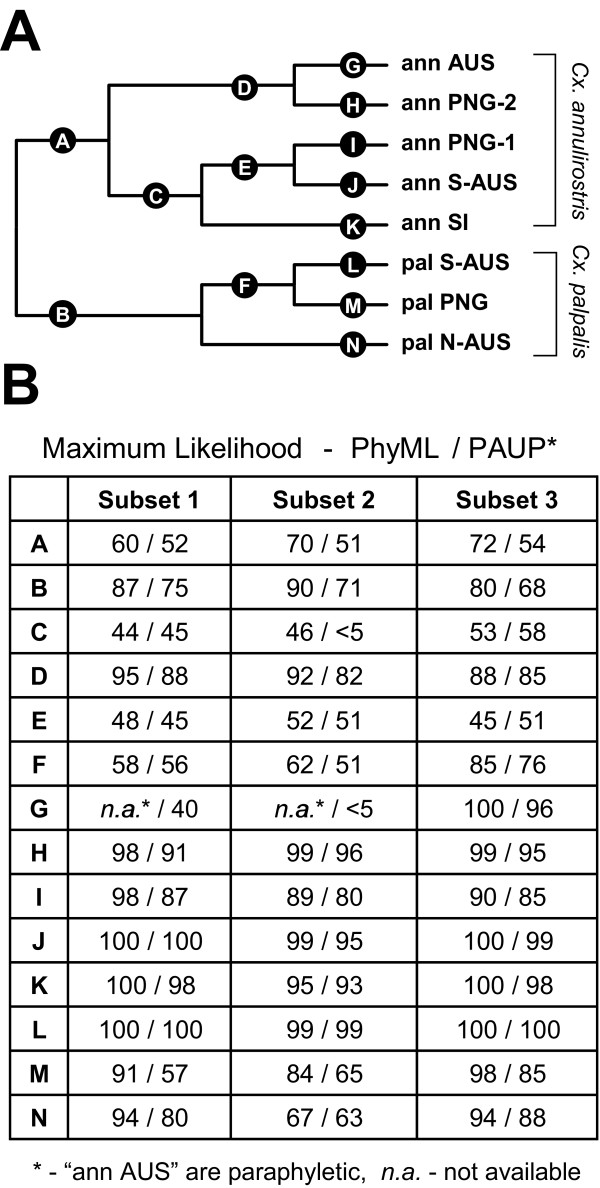
**Phylogenetic relationship of *COI *lineages**. (A) Cladogram representing lineage classification of *Cx. annulirostris *and *Cx. palpalis*. Lineage names are indicated on the right of the tree. The tree was rooted using *Cx. sitiens *and the outgroup is not shown. Maximum Likelihood bootstrap values for individual alphabetically labelled branches are summarized in Table B. (B) Three different dataset selections of 18 haplotypes from the original alignment of 182 haplotypes were used to calculate the bootstrap support; for details see Materials and Methods. Values were calculated using Maximum Likelihood in PhyML 2.4.4 (500 replicates) and PAUP* 4b10 (100 replicates); for details see Materials and Methods. Branches not supported by the analysis and dataset are indicated as * in the table and indicate paraphyly of the selected *Cx. annulirostris *ann-AUS sequences; *n.a*. indicates value not available.

Maximum Likelihood methods were used to further characterize the phylogenetic relationship by testing alternative (constrained) trees. We used three haplotype subsets and inferred constrained trees using the AU-test and the SH-test [16] to test if the constrained trees were significantly better than our best unconstrained tree (Figure 4 and Table 2). First we constructed and bootstrapped an unconstrained tree using two alternative heuristic searching algorithms; this found trees showing identical topology with marginal differences in branch support (Figure 4). The relationship of the three *Cx. palpalis *lineages was not fully resolved in the unconstrained tree and so we tested whether an alternative branching of these three lineages of *Cx. palpalis *would be rejected if compared to the unconstrained topology supporting the following: [(pal-S-AUS, pal-PNG), pal-N-AUS]. The AU-test and SH-test could not reject the alternative topologies (Table 2, Nos. I and II), indicating a trichotomy of the three lineages due to the lack of a robust phylogenetic signal.

**Table 2:** Confidence of phylogenetic tree selection using the Approximately Unbiased and Shimodaira-Hasegawa tests

		Subset 1			Subset 2			Subset 3		
Tree constraint	No.	Obs.	AU-test	SH-test	Obs.	AU-test	SH-test	Obs.	AU-test	SH-test
Unconstrained	best	-0.7	0.805	0.958	-1.4	0.887	0.974	-1.3	0.950	0.969

(pal N-AUS, pal S-AUS)	I	2.2	0.145	0.721	1.7	0.199	0.646	2.2	0.329	0.682
(pal N-AUS, pal PNG)	II	1.4	0.440	0.723	1.4	0.411	0.668	2.2	0.331	0.682
(ann S-AUS, ann AUS)	III	14.1	0.013*	0.043*	13.1	0.003*	0.035*	10.7	0.074	0.103
(ann PNG-1, ann PNG-2)	IV	17.9	0.003*	0.027*	14.7	0.002*	0.029*	11.9	0.030*	0.064
[(pal^†^, ann^¶^) ann SI]	V	0.7	0.526	0.831	2.1	0.220	0.668	1.3	0.131	0.758
(pal^†^, ann SI)	VI	3.2	0.104	0.547	2.2	0.210	0.595	2.2	0.330	0.593
[ann SI,(ann*)]	VII	2.4	0.224	0.637	1.5	0.317	0.717	1.4	0.269	0.696

Approximately Unbiased (AU) and Shimodaira-Hasegawa (SH) tests performed using CONSEL 0.1h. Obs. – test statistics; * *P*-value < 0.05, suggests that the constraints are significantly different – rejected; pal^† ^– pal PNG, pal N-AUS, pal S-AUS; ann^¶ ^– ann S-AUS, ann PNG-1, ann PNG-2, ann AUS. Abbreviations: ann – *Culex annulirostris*, pal – *Culex palpalis*; for lineage description refer to Figure 2 and 4.

Next we tested if we could reject the monophyly of the Australian lineages of *Cx. annulirostris *ann-AUS and ann-S-AUS as these two lineages appeared to be polyphyletic on the unconstrained tree (Figure 4). The AU-test and SH-test rejected this constraint in subset 1 and 2 (Table 2, Nos. III), and thus supported the separate evolutionary origin of the two Australian *Cx. annulirostris *lineages. In subset 3, this topology was not rejected although the *P*-values for both tests were low. Similarly, the monophyly of the *Cx. annulirostris *lineage ann-PNG2 with ann-PNG1 in PNG was rejected by both tests in all three subsets (Table 2, Nos. IV).

The *Cx. annulirostris *lineage ann-SI from the Solomon Islands proved problematic as its placement on the phylogenetic tree was ambiguous (Figure 4). Neither the AU-test nor the SH-test could reject the alternative placing of ann-SI: (i) as a sister to the rest of *Cx. annulirostris *with *Cx. palpalis *(Table 2, Nos. V); (ii) as a sister to the rest of *Cx. palpalis *only (Table 2, Nos. VI); or (iii) as a sister to the rest of *Cx. annulirostris *only (Table 2, Nos. VII). Hence, the evolutionary origin for the ann-SI lineage is unresolved.

## Discussion

*Culex annulirostris *and *Cx. palpalis *have overlapping morphology and are made up of several distinct *COI *lineages whose major groupings are congruent with the ITS1 diagnostic developed to separate these cryptic species [12]. *Culex sitiens *was most divergent and showed little genetic or geographic structure throughout our collection sites in Australia, PNG and Timor Leste. Its use of salt and brackish water larval habitats has allowed for the rapid exploitation of uniform coastal habitats throughout an extensive distribution that spans from Asia to Australia [6,17].

*Culex palpalis *comprises three geographically structured *COI *lineages. The branch to the pal-S-AUS lineage from southern Australia (site 10) is well supported. The pal-PNG and pal-N-AUS lineages are supported biologically in that they appear restricted to southwest PNG and the Torres Strait (pal-PNG, sites 23, 24 and 25) and to northern Australia (pal-N-AUS, sites 1, 2, 18, 20 and 22). The larval habitats of this mosquito are fresh-water sites with relatively low levels of organic matter [6].

The four major lineages of *Cx. annulirostris *indicate extended allopatric isolation, while the sublineage ann-PNG2 is a more recent event. The ann-SI lineage includes specimens from the north and south Solomon Islands and probably represents a separate Pacific island species. The ann-S-AUS lineage is more common throughout southern Australia and is found in much lower relative numbers along eastern Australia and in Torres Strait; no ann-S-AUS individuals were identified from the Northern Territory or Western Australia. The Australian lineage (ann-AUS) is the largest and most widespread, found throughout Australia and extending well into PNG with one haplotype found in Timor Leste (site 30). There is increasing evidence that ann-AUS may not have the intrinsic or extrinsic ability to be an effective JEV vector. Blood-meal studies from *Cx. annulirostris *mosquitoes collected in northern Queensland and Cape York, that would represent ann-AUS, indicate a feeding preference for marsupials, thus diverting host-seeking mosquitoes away from pigs and decreasing this lineage's efficiency as JEV vector [18]. Additionally, JEV vector competency studies suggest that ann-AUS may be a relatively inefficient laboratory vector for the JEV genotype I strain which has been present in the Torres Strait and northern Queensland since 2000 (A. F. van den Hurk, unpublished data). The ann-PNG1 and ann-PNG2 lineages exist in PNG and the Torres Strait, and though the monophyly of these two lineages is rejected, they both show a southern limit at the top of Australia's Cape York Peninsula (site 22), which correlates exactly with the current southern limit of JEV activity [5].

In light of this *COI *diversity it is not surprising that *Cx. annulirostris *is found in a wide variety of larval habitats including fresh and slightly brackish water, and in habitats with relatively high levels of organic matter as well as transient habitats [6,19,20]. If these divergent lineages represent separate biological species, it is unlikely that morphology will distinguish these taxa and another DNA-based diagnostic tool will be required to study these mosquitoes.

### Mosquito phylogeography in Australasia

The geological events of the Miocene, Pliocene and Pleistocene are believed to be responsible for considerable speciation events in this region [21,22]. There were probably two dispersal opportunities for culicid fauna to move east into the Australian region. The first was during the early Pliocene (5-3.4 mya) when the Indo-Malayan Archipelago was in place and an easterly spread of Oriental fauna was possible. The second was during the Pleistocene glaciation periods (1.6-0.01 mya), when vast areas of the Sunda shelf (joining the Malaysian and Indonesian archipelagos) and Sahul shelf (joining Australia and New Guinea) were exposed, facilitating the movement of fauna down the Indo-Malayan Archipelago [23]. Present coastlines of New Guinea and northern Australia were formed approximately 15,000-8,000 years ago and the land bridge between Australia, the Torres Strait and New Guinea was last flooded 8,000-6,000 years ago [23,24].

We suggest two different biogeographic histories for these *Culex *taxa. *Culex annulirostris *and *Cx. palpalis *have distributions limited mostly to Australasia, with genetic and geographic structure suggestive of an extended evolutionary history in this region allowing the evolution of multiple divergent lineages. Thus *Cx. annulirostris*, *Cx. palpalis *or an ancestor thereof appeared during the early Pliocene (5-3.4 mya), which would have permitted the necessary evolutionary time for geographic expansion and the development of divergent lineages. Since *Cx. sitiens *displays little genetic or geographic structure in Australia and has a distribution spanning Australasia and Southeast Asia to as far north as India [17], we suggest a more recent expansion into Australasia, probably during one of the more recent Pleistocene glaciations (1.6-0.01 mya).

### Barcoding closely related Culex taxa

The mtDNA *COI *sequence has been assessed as a marker for evaluating mosquito diversity and has proved useful in DNA barcoding studies of mosquitoes across several genera and species identified through conventional morphological taxonomy [25,26]. In insect molecular systematics, other markers have been used with success: *18S *rDNA, *28S *rDNA, *NADH dehydrogenase subunit 5 *and *cytochrome oxidase II *[27,28]. Here we have used the *COI *marker on closely related *Culex *mosquito taxa that were genetically identified to species using the ITS1 [12]. We employed a Maximum Likelihood framework for hypothesis testing [16] that provided insights into the evolutionary history of these three taxa. As both ITS1 markers and *COI *concurred with the latter, revealing several more divergent lineages within these taxa, we advocate that *COI *is a reasonable starting point for molecular identification of potential cryptic mosquito species of the genus *Culex*.

## Conclusion

The different geographic distributions of these distinct lineages suggest biological variety and thus it is reasonable to assume that many of these lineages will have different abilities to transmit endemic and exotic arboviruses. The coincidental southern limit of ann-PNG1, ann-PNG2 and JEV activity presents a plausible hypothesis as to why JEV has not established on the Australian mainland, despite the apparent abundance of local *Cx. annulirostris *vectors and feral pig amplifying hosts [3]. As stressed above, the endemic ann-AUS lineage may not be an effective JEV vector due to its local marsupial feeding preferences and relative inefficiency in transmitting the JEV genotype I strain and thus may provide northern Australia with a natural buffer zone [18]. If either ann-PNG1 or ann-PNG2 is responsible for this JEV activity it is important to determine their species status and monitor their distributions to determine if they are moving south into Australia. If these lineages are not separate species then there is the possibility for gene flow from the PNG lineages to the ann-AUS lineage on the Australian mainland, and this may enhance the JEV potential of local ann-AUS populations. Analysis of the vector competency of individual lineages is now required to characterize their intrinsic ability to transmit JEV, while nuclear DNA studies on sympatric populations would establish if natural mating barriers exist.

The question must now be asked as to whether ann-PNG1 and ann-PNG2 are exotic taxa naturally expanding their range from southwest PNG into Australia – or are we observing the effects of climate change, such as have been suggested for the spread of the bluetounge arbovirus through Europe alongside the northward movement of its *Culicoides *vectors [29]. In Australia, rapid shifts of latitude clines in *Drosophila *over the last 20 years have provided evidence for biological changes through regional warming in Queensland [30], and this may also facilitate the expansion of independently mobile fauna from New Guinea.

## Methods

### Specimen collection and identification

Mosquitoes were collected from 43 sites representing 30 map locations in Australia, PNG, Timor Leste and the Solomon Islands (Bougainville and Guadalcanal) (see Figure 1, see Additional file 1 and 2). Adult mosquitoes were collected using CO_2_-baited encephalitis virus surveillance (EVS) traps with and without 1-octen-3-ol (octenol). Specimens were morphologically identified using the keys of Lee et al. [6] and Marks [31]. Mosquitoes were stored in liquid nitrogen, dry ice, on silica gel, or in 70% ethanol prior to total DNA extraction. Colony *Cx. annulirostris *from the Army Malaria Institute (Brisbane, Queensland, Australia) were used as reference material. Total DNA was extracted from mosquitoes using a salt extraction and ethanol precipitation procedure [12]. Due to problems with adult morphology, all material was genetically identified to species by an ITS1 PCR-RFLP procedure previously developed to discriminate between *Cx. annulirostris, Cx. palpalis *and *Cx. sitiens *[12].

### Genetic analysis COI amplification

A 538 bp 5' fragment of the *COI *gene was PCR amplified and sequenced using the same primer pair for amplification and sequencing; F-COI50 (5'-GTA GTT TAG TAG AAA ATG GAG C-3') and R-COI650 (5'-TAG CAG AAG TAA AAT AAG CTC G-3'). Reactions of 25 μl contained 2.5 mM MgCl_2_, 200 pM for each dNTP, 0.6 unit *Taq *(Fisher Biotech, WA, Australia), and approximately 1–10 ng of genomic DNA template (~1 μl). The cycling was as follows; denaturing at 94°C for 3 min followed by 35 cycles of 94°C for 1 min, 48°C for 1 min, 72°C for 1 min, and a final elongation for 3 min at 72°C. The correctly sized PCR product was verified on agarose gel and the remainder purified using a QIAquick PCR purification kit (Qiagen). Individual PCR products were directly sequenced in both directions at the Australian Genome Research Facility (University of Queensland, Brisbane, Australia). Haplotype a126 was PCR amplified and sequenced three times to confirm its unique amino acid substitution. Sequences have been deposited in GenBank [GenBank: DQ673677–DQ673858].

### COI sequence analysis

Individual sequences were assembled with Sequencer 4.2.2 (GeneCodes, MI, USA). Nucleotide and haplotype diversity within the lineages identified was calculated using DNASP 4.10 [32]. Identical haplotypes were pooled and only unique haplotypes were used for further analysis. Composition of the nucleotide sequences was analyzed using MEGA 3.1 [33]. Sequence divergences were calculated using the Kimura 2 parameter distance model using MEGA 3.1 [33]. The maximum of intra-population distance and the minimum of inter-population distance were calculated using MEGA 3.1. We applied a 3% threshold to represent different categories of "species" [14].

A multiple sequence alignment was constructed comprising 182 unique haplotypes from 208 *Cx. annulirostris*, 37 *Cx. palpalis *and 28 *Cx. sitiens *(see Additional file 3). Haplotypes of *Cx. sitiens *served as an outgroup. The alignment consisted of 538 nt coding for 179 amino acids using the invertebrate mitochondrial code (Supplementary Alignment A1). The nucleotide sequence alignment was analyzed using Maximum Likelihood with the GTR+Γ+I model using PhyML 2.4.4 [34]. The robustness of the Maximum Likelihood tree was evaluated by the bootstrapping method with 500 replicates using PhyML. For the nucleotide model selection we employed a hierarchical likelihood ratio test (LRT) as well as the Akaike information criterion (AIC) implemented in ModelTest 3.6 [13] in cooperation with PAUP* 4b10 [35].

In addition, the dataset was analysed by Bayesian phylogenetic analysis using MrBayes 3.1.2 [36]. We took advantage of MrBayes' ability to relax the parameters of the nucleotide model over subsets of the alignment, in order to better model the nucleotide evolution – particularly at different nucleotide coding positions [36]. A covarion model was used to better model the between-lineage variability [37].

Initially the alignment was divided into partitions based on coding position. Then we unlinked the parameters (shape, revmat, statefreq) between these partitions and applied a 4× 4 nucleotide model. Finally, for the 1st and 2nd codon positions we used the model with only one substitution category with all rates equal (F81 model), and for the 3rd codon position we used the model with 6 categories with a gamma shape parameter plus invariants including the covarion parameter (GTR+Γ+I+cov model). Metropolis-coupled Markov chain Monte Carlo analyses were run with one cold and three heated chains (temperature set to default 0.2) for 5,000,000 generations and sampled every 200 generations. This process was performed three times from a random starting tree and ran well beyond convergence. Trees before convergence were discarded for the reconstruction of the consensus Bayesian tree with posterior probabilities.

### Alternative tree topology testing using haplotype subsets

To further address the phylogenetic relationship within the obtained haplotypes, we restricted the alignment into three subsets, each comprising 18 haplotypes (10 haplotypes from *Cx. annulirostris*, 6 from *Cx. palpalis *and 2 from *Cx. sitiens *which served as an outgroup). Given that Maximum Likelihood calculations with all haplotypes were prohibitively time-consuming, these subsets served as surrogate abstractions of the full dataset. For each lineage unit we selected two haplotypes based on the following approach: Subset 1 – an ancestor haplotype and its closest haplotype (a44, a51, a89, a92, a98, a103, a117, a119, a128, a132, p12, p25, p28, p29, p30, p33, s7, s8); Subset 2 – two haplotypes from network extremities (a51, a64, a89, a93, a98, a99, a119, a107, a128, a131, p10, p20, p25, p30, p32, p33, s9, s8); and Subset 3 – an ancestor and an extremity of the network (a64, a66, a88, a93, a99, a102, a107, a113, a130, a131, p5, p10, p20, p22, p31, p32, s4, s9). Calculations on the three subsets enabled us to map the variability of the haplotype phylogenies. For each subset we calculated a Maximum Likelihood tree with bootstraps and applied a variety of constraints to test both our hypothesis and the robustness of the optimal tree.

The best trees and all constraints were inferred in PAUP*4b10 [35]. We first reconstructed a Neighbour joining tree and used it to estimate the GTR+Γ+I likelihood parameter. Then the parameters were fixed and used in the inference of a Maximum Likelihood tree using a heuristic search with 20 random sequence additions with NNI swapping. The resulting GTR+Γ+I model parameters were used for the Maximum Likelihood tree reconstruction for the unconstrained tree as well as for all constrained trees. For the unconstrained trees we calculated bootstraps using 100 replicates. Site likelihoods for individual trees were calculated using PAUP*4b10 [35] and these were used for the Approximately Unbiased (AU) test [16] and the Shimodaira-Hasegawa (SH) test [38], both implemented in CONSEL 0.1 h [39]. A value of *P*>0.05 was considered statistically significant to reject the hypothesis that the two trees were significantly different.

## Abbreviations

PNG, Papua New Guinea; *COI*, *cytochrome oxidase subunit I gene*; JEV, Japanese encephalitis virus; ITS1; internal transcribed spacer 1

## Authors' contributions

NWB conceived and funded the project. SH performed the experiments. SH and JS performed the analysis. AFH, RDC, PIW, CAJ and RCR provided field-collected material. NWB, SH and JS wrote the paper. All authors read and approved the final manuscript.

## Supplementary Material

Additional File 1 Full data set including morphology, PCR identification, haplotypes of mosquitoes analyzed and GenBank accession numbers. Click here for file

Additional File 3 Multiple sequence alignment of 182 haplotypes obtained in this study presented in PHYLIP format. Click here for file

Additional File 2 List of *Culex annulirostris*, *Cx. palpalis *and *Cx. sitiens *haplotypes obtained in this study. Click here for file
